# Activity and levels of TNF-α, IL-6 and IL-8 in saliva of children and young adults with dental caries: a systematic review and meta-analysis

**DOI:** 10.1186/s12903-024-04560-8

**Published:** 2024-07-18

**Authors:** Mario Alberto Alarcón-Sánchez, Julieta Sarai Becerra-Ruiz, Anna Avetisyan, Artak Heboyan

**Affiliations:** 1 Biomedical Science, Faculty of Chemical-Biological Sciences, Autonomous University of Guerrero, Chilpancingo de los Bravo, Guerrero, 39090 Mexico; 2 Institute of Research of Bioscience, University Center of Los Altos, University of Guadalajara, Tepatitlán de Morelos, Jalisco, 47600 Mexico; 3 Department of Therapeutic Stomatology, Faculty of Stomatology, Yerevan State Medical University after Mkhitar Heratsi, Str. Koryun 2, Yerevan, 0025 Armenia; 4 Department of Research Analytics, Saveetha Institute of Medical and Technical Sciences, Saveetha Dental College and Hospitals, Saveetha University, Chennai, 600 077 India; 5 Department of Prosthodontics, Faculty of Stomatology, Yerevan State Medical University after Mkhitar Heratsi, Str. Koryun 2, Yerevan, 0025 Armenia; 6 Department of Prosthodontics, School of Dentistry, Tehran University of Medical Sciences, North Karegar St, Tehran, Iran

**Keywords:** Tumor necrosis factor- alpha, Interleukin-6, Interleukin-8, Saliva, Dental caries, Biomarkers, Systematic review

## Abstract

**Background:**

Cytokines play an important role in the immunopathogenesis of dental caries. A systematic review and meta-analysis was carried out with the following three objectives: 1)To deepen and discuss through a comprehensive analysis of the literature the effects of dental caries on the activity and levels of TNF-α, IL-6 and IL-8 in saliva of children and young adults, 2)To compare the levels of this cytokines in saliva of the exposure group (moderate-severe dental caries) with the control group (caries-free or mild dental caries), and 3)To determine whether the levels of these cytokines could be used as a complementary clinical diagnostic tool to assess the severity of dental caries.

**Methods:**

The protocol followed PRISMA and Cochrane guidelines and was registered in the Open Science Framework (OSF): 10.17605/OSF.IO/MF74V. A digital search was performed in PubMed/MEDLINE, Cochrane, Scopus, and Google Schoolar databases from February 15th, 2012, to January 13th, 2024. The methodological validity of the selected studies was assessed using Joanna Briggs Institute (JBI) tool. A meta-analysis was performed using a random-effects model to evaluate the association between dental caries/health, and the concentration of TNF-α, IL-6 and IL-8.

**Results:**

The search strategy provided a total of 126 articles, of which 15 investigations met the inclusion criteria. The total number of patients studied was 1,148, of which 743 represented the case/exposure group, and 405 represented the control group. The age of the patients ranged from 3 to 25 years. IL-6 was the most prevalent cytokine in the saliva of children and young adults with active dental caries. The meta-analysis revealed that there are significant differences between the levels of IL-6 and TNF-α in saliva of children with active dental caries compared to their control groups.

**Conclusions:**

The findings suggest that IL-6 and TNF-α levels may have potential as complementary biomarkers for assessing dental caries severity. However, further research is needed to validate these findings in larger and more diverse populations before clinical application.

## Background

Dental caries (DC) is a chronic, non-transmissible, infectious disease with a high prevalence worldwide [1]. In fact, it is estimated that approximately 2.8 billion people worldwide are affected by this pathological entity [2]. According to the latest reports presented by the World Health Organization (WHO), the proportion of cases of untreated DC in the first dentition (in children) is 42.7%, while, in the permanent dentition (in adults) it is 28.7% [3]. Therefore, it has become a recognized public health challenge, with a serious economic and health burden that predominantly affects the most vulnerable groups, which are low-income individuals belonging to ethnic or racial minorities [4]. In turn, this condition can also negatively affect oral health-related quality of life (OHRQoL) in the general population [5].

DC is influenced by genetic factors such, as single nucleotide polymorphisms (SNPs), and environmental factors such as smoking and nutritional deficiencies. A primary cause is the prolonged exposure of the teeth to a biofilm constituted mainly by virulent bacteria, such as *Streptococcus*, *Lactobacillus* and *Actinomyces* species, which adhere on the enamel surface and produce acids by metabolizing dietary carbohydrates gradually demineralizing its structure [6, 7]. Thus, upon bacterial challenge and recognition, odontoblasts, pulp tissue fibroblasts, and immune cells such as dendritic cells, macrophages and neutrophils cooperatively induce a multitude of molecules, among them cytokines and chemokines such as interleukin-1 beta (IL-1β), tumor necrosis factor alpha (TNF-α), interleukin-6 (IL-6), interleukin-8 (IL-8) and prostaglandins that prolong the inflammatory state, in turn favoring the activation of the innate and adaptive immune response [8]. Among their multiple functions, these cytokines regulate mechanisms such as recruitment (chemotaxis) and extravasation of phagocytes to the site of inflammation and destruction, they also promote cell activation and differentiation; for example, they induce osteoclastogenesis by up-regulating the receptor of nuclear factor κB receptor ligand (RANKL) and on the other hand they also contribute to the differentiation of T helper (Th) cells into different subsets such as follicular Th cells and Th17 cells [6, 8, 9].

The production of antibodies by B cells is another effect produced by these molecules [9, 10]. Scientific evidence has shown that the levels of TNF-α, IL-6, and IL-8 are altered in the saliva of subjects with different oral pathologies such as DC, oral lichen planus, periodontitis, peri-implantitis, primary Sjögren’s syndrome, oral leukoplakia, and drug-related osteonecrosis of the jaw, among others [11]. In relation to DC, numerous studies have been published evidencing differences in the concentrations of these inflammatory mediators particularly in the saliva of children and young adults with carious lesions at a moderate to severe stage (exposure group), compared to those individuals free of caries or mild caries (control group), suggesting that these cytokines may play a role in the pathogenesis of the disease and could be investigated further as potential biomarkers for the diagnosis and follow-up of DC [12–26]. Therefore, it is a topic that deserves further research. Finally, it is important to mention that, if DC is not treated in time, it progresses and invades the pulp tissue causing pain with the consequent formation of granulomas and dental abscesses [27]. Up to this point, the infection continues to be local, however, if not stopped, it can spread systemically and produce a serious infection that can even lead to death [28].

The diagnosis of DC is based on strict clinical visual and radiographic evaluation; however, these tools alone cannot predict the onset, much less monitor the progression of carious lesions [29]. In this regard, in recent years, researchers have employed new biochemical and molecular diagnostic tools in the oral environment contributing to the study of Omics sciences, such as Genomics and Epigenomics, Transcriptomics, Proteomics, Metabolomics, and the Microbiome of different biological samples such as gingival crevicular fluid, gingival tissue, dentobacterial plaque, and saliva [30]. These areas of study are complementary to traditional methods and are carried out by analyzing different biomarkers [31]. A biomarker corresponds to a molecule that is able to discriminate between a state of health or disease [32]. Thus, one of the biofluids that is in frequent contact with teeth, is abundant, easy to collect, painless and also plays an important role in the development of DC; it is saliva [33]. Saliva corresponds to a viscous, foamy, and milky liquid, very versatile, which fulfills functions such as lubrication and protection of the different surfaces that constitute the oral cavity, cleaning of these surfaces, buffering capacity, formation of the acquired salivary film, mineralization of the teeth and also has antimicrobial effects [34, 35]. Other functions include tissue repair, it is also involved in taste, chewing, initial digestion, bolus formation, swallowing and speech articulation [36, 37]. Saliva consists of 94–99% water, the remainder (1–6%) is made up of organic matter such as glycoproteins, cytokines, chemokines, and immunoglobulins (Ig), antimicrobial peptides, hormones, antioxidants, lipids, nucleic acids, exfoliated cells and neutrophils, as well as bacteria [38]. The inorganic component includes sodium, potassium, calcium, magnesium, bicarbonate, and phosphate ions [39]. For such reason, saliva is enriched in biomarkers and the area of study that explores their composition and importance in health conditions or disease is salivaomics, a very promising discipline [40].

According to previous studies [39–43] only systematic reviews have been published exploring the concentrations of protein biomarkers such as malondialdehyde, superoxide dismutase, uric acid, alpha-amylase, proline-rich proteins, histatin-5, lactoperoxidase, mucin-1, carbonic anhydrase VI, proteinase-3, secretory IgA, and staterin in the exposure group compared to the control group. However, this is the first time a systematic review and meta-analysis has been written on the subject.

Therefore, the objectives of the present study were as follows:

1. To deepen and discuss by means of a comprehensive review of the literature the effects of DC on the activity and levels of TNF-α, IL-6 and IL-8 in saliva of children and young adults.
2. To compare the levels of TNF-α, IL-6 and IL-8 in saliva of the exposure group with the control group.
3. To determine whether the levels of these cytokines could be used as a complementary clinical diagnostic tool to assess the severity of DC.

## Methods

### Protocol and registration

For this review work, followed the Preferred Reporting Items for Systematic Review and Meta-Analysis (PRISMA) [44], and Cochrane Handbook for Systematic Reviews guidelines [45]. Additionally, the protocol was recorded with the Open Science Framework (OSF) enrollment: 10.17605/OSF.IO/MF74V.

### Research question

Are there changes in the levels of TNF-α, IL-6 and IL-8 in saliva of children and young adults with active DC compared to their controls groups?

### Eligibility criteria

The PECOS design was used to assess eligibility of the document.

- Population (P): Systemically healthy children and young adults.
- Exposure (E): Levels of TNF-α, IL-6 and IL-8 in saliva.
- Comparator (C): Children or young adults with active DC/high caries (exposure group), and children or young adults without caries/low caries (control group).
- Outcome (O): Differences in the levels of TNF-α, IL-6 and IL-8 in saliva of the exposure group with respect to the control group.
- Study design (S): Clinical studies: Cross-sectional, cohort and case-control studies.

Only articles reporting the exact values of cytokines levels in saliva of both study groups either individually or together were included. On the other hand, the following exclusion criteria were established: (1)adults older than 25 years; (2)children with physical or intelectual disabilities; (3)articles that did not use a predefined index to assess DC among study groups; (4)quantification of TNF-α, IL-6 and IL-8 in gingival crevicular fluid, serum or gingival tissue; (5)articles in a language other than english; (6)articles published before 2012; (7)case reports or series; (8)comprehensive, narrative or scoping reviews; (9)systematic reviews and meta-analysis; (10)animal studies; and (11)book chapters.

### Search strategy and study selection

We searched in PubMed, Cochrane, Scopus, and Google Scholar data bases for articles published from February 15th, 2012, to January 13th, 2024, using the same focus question for this research. In addition to the electronic search, a manual search was performed in the following journals: *Odontology*, *Journal of Dentistry*, *Journal of Prosthodontics-Implant Esthetic and Reconstructive Dentistry*, *European Journal of Pediatric Dentistry*, *Journal of Adhesive Dentistry*, *Journal of Esthetic and Restorative Dentistry*, *Pediatric Dentistry*, and *Journal of Clinical Pediatric Dentistry*. Table 1 shows the search strategy used for the selection of articles in the different search engines.

**Table 1:** The full search strategy used in the PubMed, Scopus, Google Schoolar, and Cochrane database

Database	Search Strategy
PubMed	((“Dental Caries“[Mesh]) AND “Saliva“[Mesh]) AND) AND “Cytokines“[Mesh]
Cochrane, Scopus, and Google Schoolar,	TITLE-ABS-KEY (Dental Caries AND Salivary AND Tumor Necrosis Factor alpha OR Interleukin-6 OR Interleuin-8)

Once the articles were retrieved, two investigators (M.A.A.S and J.S.B.R) independently evaluated the titles and abstracts of each of the studies following the eligibility criteria to identify potentially relevant publications focused on the research topic. Any disagreement between the reviewers was discussed and resolved by reaching an agreement with a third investigator (A.H), thus excluding all irrelevant publications. Finally, the full texts were carefully evaluated and those studies that met the inclusion criteria were selected.

### Data collection

Data extraction was performed manually, reviewing and selecting the information of interest in each article. All extracted information was summarized in a Microsoft Excel sheet and evaluated independently by two reviewers (M.A.A.S and J.S.B.R). Disagreements were resolved by discussion with a third investigator (A.H) until consensus was reached. The following information was extracted from the selected research: first author, year of publication, country, type of study, number of cases, which corresponds to the exposure group and number of controls, total study population, age, gender, type of caries index used, type of saliva analyzed, method and time period in which saliva collection was carried out, amount of saliva collected, type of immunological marker evaluated, type of immunoassay, mean or median value of the concentration of the protein of interest, *p* value and main results.

### Risk of bias assessment

The quality of the cross-sectional and cohort studies was assessed using the Joanna Briggs Institute (JBI) critical appraisal tool (https://jbi.global/critical-appraisal-tools).

Scores of *≥* 7 or more were classified as “low” risk of bias, scores of 4–6 as “moderate” risk, and scores of 1–3 or less as “high” risk.

### Statistical analysis

Statistical analysis was run with STATA V.17 software (Stata Corp, College Station, TX, USA) for the construction of the meta-analysis on TNF-α, IL-6 and IL-8 levels assessed in pg/ml, between both study groups (exposure vs. control group). The standardized mean difference (SMD) with 95% confidence intervals (CI) was calculated using a random and/or fixed effects model according to the significance of the heterogeneity value (> 50%= high heterogeneity), which was estimated using the Q statistic and quantified with the (I^2^) statistic. A p* value < 0.05 was considered statistically significant. Forest plots were constructed to visualize estimates with 95% CI. Egger linear regression and funnel plot were used to assess publication bias.

## Results

### Study selection

**Fig. 1:**
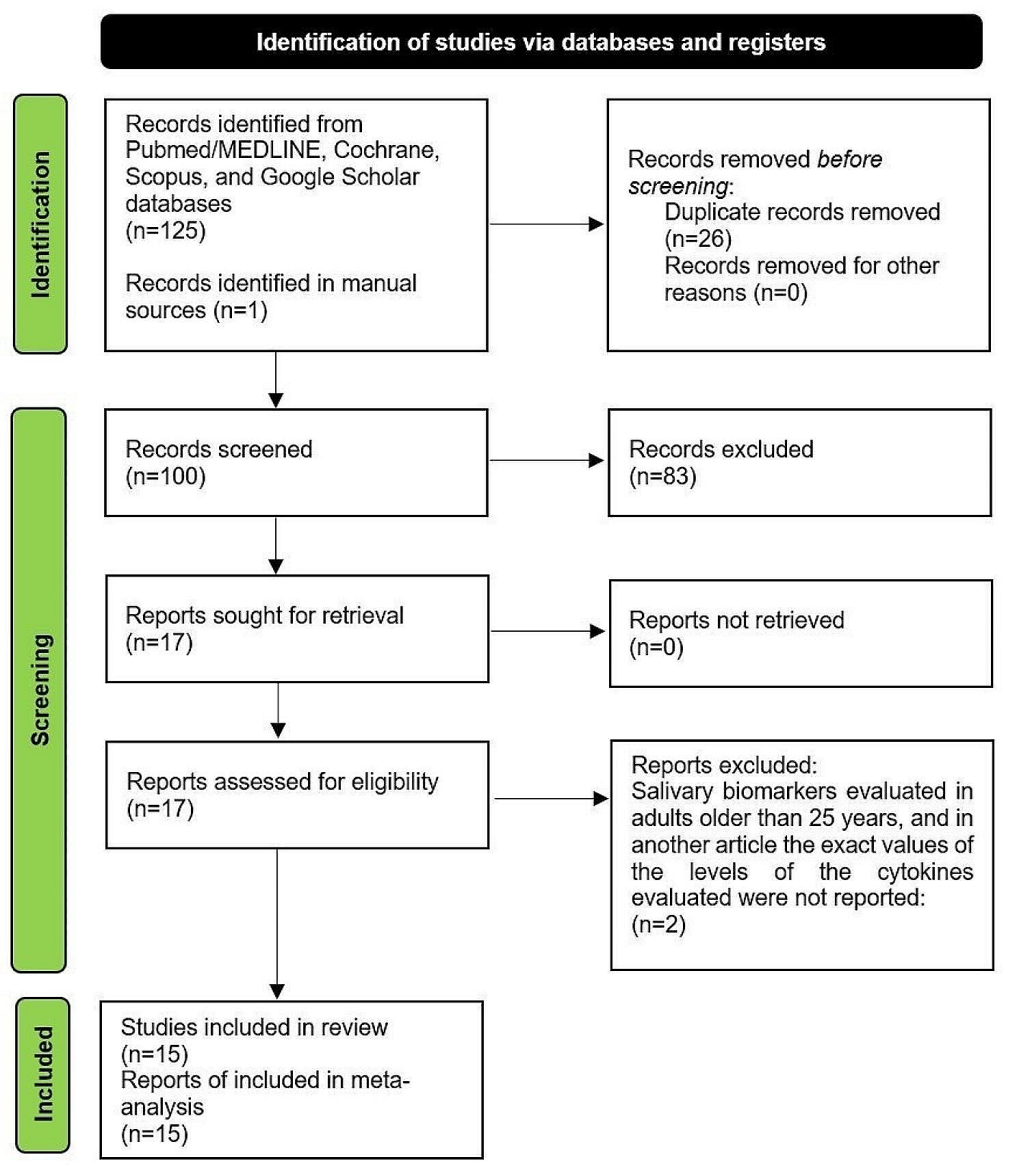
PRISMA flow diagram. PRISMA: Preferred Reporting Items for Systematic and Meta-Analyses

Initially 126 articles were found in the four databases, including PubMed (18 articles were found), Cochrane (35 were found), Scopus (25 articles were found), Google Scholar (47 articles were found), and hand searching (1 article was found). Duplicates were removed and, based on title and abstract, the remaining 100 studies were reviewed. After analyzing the full text of the remaining articles, 83 records were excluded as irrelevant. A total of 17 articles were assessed for eligibility, of which 2 studies were excluded because one of them evaluated salivary markers in adults older than 25 years and another article did not report the exact numbers of cytokine levels evaluated. Therefore, a total of 15 articles were included for the qualitative and quantitative analysis of the present review. Details of the study selection are shown in Fig. 1.

### Characteristics of the included studies

Fourteen articles with a cross-sectional design [12, 14–26] and one cohort study [13] were reviewed in this study. The total number of children and young adults studied in the included investigations was 1,148, of which 743 represented the case/exposure group (individuals with moderate-severe active DC) and 405 represented the control group (caries-free or mild caries individuals). The ages of the patients ranged from 3 to 25 years; the mean age ± standard deviation of the patients studied was 8.46 ± 6.90 years, of which 33.33% were male, 31.41% were female and in 35.26 the gender was not specified. Most of the articles were published after 2017 (13:86.66%) [12–24]. The oldest study was from 2012 [26], whereas, the most recent was from 2024 [12]. Three (20.0%) studies were conducted in India [12, 17, 24] and Iran [13, 21, 25], two (13.3%) studies in Brazil [14, 23] and Iraq [18, 19], and other studies (6.6%) in Norway [15], Switzerland [16], Mexico [20], Italy [21] and Poland [26] (Table 2).

**Table 2:** Characteristics of 14 studies included in the qualitative and quantitative analysis

Author’s & Year	Country	Study type	*n*		*n* Total	Age; mean or range (SD)	Gender F/M	Caries index	Saliva type	Saliva collection technique	Saliva collection time	Amount of saliva
			EG	CG								
Nireeksha et al., 2024 [12]	India	CS	190	70	260	18–25	Unclear	DMFT	Unstimulated saliva	NI	10am-11am	5mL
Biria et al., 2023 [13]	Iran	Cohort	25	25	50	3.93	22/28	DMFS	Unstimulated saliva	Passive drooling, 5 min	9am-11am	1mL
Leme et al., 2022 [14]	Brazil	CS	29	21	50	4.72	Unclear	Visual detection method	Unstimulated saliva	NI	NI	NI
Børsting et al., 2022 [15]	Norway	CS	137	39	176	4.37	85/91	DMFT	Stimulated saliva	Chewing paraffin, 30s	NI	4mL
Rinderknecht et al., 2022 [16]	Switzerland	CS	38	39	77	7.8	36/41	Visual detection method	Stimulated saliva	Chewing paraffin, 2–5 min	9am-11am	3.2mL
Prasanna et al., 2022 [17]	India	CS	13	13	26	NI	NI	Index WHO	Unstimulated saliva	Spitting	NI	NI
AL-Dahhan et al., 2021 [18]	Iraq	CS	8	8	16	*≤* 10	NI	Visual detection method	Unstimulated saliva	Spitting, 10 min	9am-11am	NI
Mohammed et al., 2021 [19]	Iraq	CS	50	46	96	20.73	49/51	DMFS	Unstimulated saliva	Spitting, 5 min	9am-11am	1.3mL
Ramírez-De los Santos et al., 2020 [20]	Mexico	CS	38	5	43	5.8 / 3–8	15/28	ICDAS	Unstimulated saliva	Spitting	NI	NI
Lo Giudice et al., 2019 [21]	Italy	CS	39	20	59	4–16	NI	DMFT	Unstimulated saliva	Spitting, 10 min	10am-11am	NI
Nazemisalman et al., 2018 [22]	Iran	CS	45	34	79	3–18	49/30	Visual detection method	Unstimulated saliva	Spitting, 15 min	9am-12pm	4mL
Ribeiro et al., 2017 [23]	Brazil	CS	20	20	40	3.65	NI	DMFT	Unstimulated saliva	Chewing paraffin, 5 min	NI	NI
Sharma et al., 2017 [24]	India	CS	25	25	50	4.3	23/27	DMFT	Unstimulated saliva	Passive drooling, 10 min	9am-11am	2mL
Seyedmajidi et al., 2015 [25]	Iran	CS	60	30	90	3–5	NI	DMFS	Unstimulated saliva	Spitting	10am	4mL
Gornowicz et al., 2012 [26]	Poland	CS	26	10	36	18	NI	DMFT	Unstimulated saliva	Spitting, 10 min	9am-11am	NI
**Summary of variables included in the study** →			743	405	1,148	8.46(6.90)	279/296	DMFT (35.71%)	US (86.66%)	Spitting, 8 min (57.14%)	9am-11am (42.85%)	3.89mL

Abbreviations: CS: Cross-Sectional study, EG: Exposure group, CG: Control group, SD: standard deviation, DMFS: Decayed, Missing, and Filled Surfaces, DMFT: Decayed, Missing, Filled, Teeth, ICDAS: International Caries Detection and Assessment System, WHO: World Health Organization, NI: No information, US: Unstimulated saliva

Six (40%) [12, 15, 21, 23, 24, 26] studies used the Decayed, Missing, Filled, Teeth (DMFT) as the caries detection index, followed by the visual method of detection (28.5%) [14, 16, 18, 22]. The most frequently collected saliva type was unstimulated saliva (85.7%) [12–14, 17–26] and the most frequent collection technique was by Spitting (57.1%) [17–22, 25, 26] for an average time of 8 min. In addition, most studies (42.8%) [13, 16, 18, 19, 24–26] indicated that between 9am and 11am saliva collection was performed. On average, 3.89 mL of saliva was collected (Table 2).

The most frequently evaluated marker was IL-6 (73.3%) [12, 14–20, 22, 23, 25], followed by TNF-α [14, 16, 18, 22–24, 26] and IL-8 (46.7%) [13, 15, 16, 20, 24–26]. Among the 15 included studies, 11 (73.3%) [12, 13, 17–22, 24–26] used the enzyme-linked immunosorbent assay (ELISA) for the determination of inflammatory mediator levels, while the remaining 26.6% [14–16, 23] used the multiplex assay. Table 3 shows the different brands of kits used, the levels of cytokines reported and the main results obtained (Table 3).

**Table 3:** Comparison of biomarkers between cases and controls in 14 studies

Author’s & Year	Marker type	Protein evaluation method	Marker value (SD) in exposure group	Marker value (SD) in control group	*p*-Value	Main comments
Nireeksha et al., 2024 [12]	IL-6	ELISA (Booster Biologicals)	IL-6 (pg/mL) 31.15(40.98)	IL-6 (pg/mL) 28.33(31.81)	0.813	↑ IL-6 levels in group with DC compared to control group
Biria et al., 2023 [13]	IL-8	ELISA (MyBioSource)	IL-8 (pg/mL) 35.84(4.39)**	IL-8 (pg/mL) 20.00(3.88)*	< 0.001*	↑ IL-8 levels in group with DC compared to control group Plaque index (*p 0.018), and duration of night feeding with Breast milk or formula (*p 0.021) had a significant influence on development of severe caries
Leme et al., 2022 [14]	TNF-α	Multiplex assays (System-Merck)	TNF-α (pg/mL) 7.00(23.00)	TNF-α (pg/mL) 5.00(10.00)	> 0.05	↑TNF-α levels in group with DC compared to control group
Børsting et al., 2022 [15]	IL-8, IL-6	Multiplex assays (Olink Bioscience)	IL-8 (pg/mL) 12.44(0.88) IL-6 (pg/mL) 4.58(1.75)	IL-8 (pg/mL) 12.28(0.90) IL-6 (pg/mL) 4.18(1.97)	NS	↑ IL-8, and IL-6, levels in group with DC compared to control group
Rinderknecht et al., 2022 [16]	IL-8, IL-6, TNF-α	Multiplex assays (Millipore)	IL-8 (pg/mL) 297(187,615) IL-6 (pg/mL) 3.1(1.4,8.6) TNF-α (pg/mL) 6.7(3.9,15.1)	IL-8 (pg/mL) 265(132,585) IL-6 (pg/mL) 2.9(1.4,7.1) TNF-α (pg/mL) 8.1(4.5,12.9)	0.30 0.61 0.40	↑ IL-8 and IL-6 levels in group with DC compared to control group It was shown that, IL-6 has an increasing trend in children per year (*p* 0.093). Furthermore, the concentration of TNF-α and IL-6 was slightly higher in boys than in girls
Prasanna et al., 2022 [17]	IL-6	ELISA (BioLegend MAX™)	IL-6(pg/mL) 3.53	IL-6(pg/mL) 4.05	0.858	A slight non-significant increase in IL-6 levels was observed in the caries-free group compared to the caries group
AL-Dahhan et al., 2021 [18]	IL-6, TNF-α	ELISA (Elabscience)	IL-6(pg/mL) 13.9 TNF-α (pg/mL) 4.05	IL-6(pg/mL) 57.1 TNF-α (pg/mL) 3.5	NI	↓ IL-6 levels in group with DC compared to control group ↑TNF-α levels in group with DC compared to control group
Mohammed et al., 2021 [19]	IL-6	ELISA	IL-6(pg/mL) 14(0.914)**	IL-6(pg/mL) 3.07(0.36)*	< 0.05*	↑ IL-6 levels in group with DC compared to control group Negative correlations between IL-6 with IL-10 (-0.058), pH (-0.010), Malondialdehyde (-0.444), and flow rate (-0.314) (*p < 0.05)
Ramírez-De los Santos et al., 2020 [20]	IL-8, IL-6	ELISA (BioLegend)	IL-8 (pg/mL) 183.90(123.35–235) IL-6 (pg/mL) 7.65(3.93–23.81)**	IL-8 (pg/mL) 200(198.95–267) IL-6 (pg/mL) 2.04(1.78–6.23)*	0.152 < 0.05*	↓ IL-8 levels in group with DC. ↑ IL-6 levels in group with cavited caries lesions (ICDAS-II code 3–6), compared non-cavited caries lesions (ICDAS-II code 0–2). Slight increase in IL-8 levels non-cavited caries lesions compared cavited caries lesions group.
Lo Giudice et al., 2019 [21]	IL-6	ELISA (R&D Systems)	IL-6 (pg/mL) 30.2(11.8)**	IL-6 (pg/mL) 19.02(5.3)*	< 0.001*	↑ IL-6 levels in group with DC compared to control group
Nazemisalman et al., 2018 [22]	TNF-α	ELISA (IBL)	TNF-α (pg/mL) 35.20(16.23)**	TNF-α (pg/mL) 26.20(6.25)*	< 0.001*	↑ TNF-α levels in group with DC compared to control group
Ribeiro et al., 2017 [23]	IL-6, TNF-α	Multiplex assays (Millipore)	IL-6(pg/mL) 8.61(7.84) TNF-α (pg/mL) 4.02(1.44)	IL-6(pg/mL) 4.24(1.68) TNF-α (pg/mL) 3.79(1.71)	NI	↑ IL-6, and TNF-α levels in group with DC compared to control group
Sharma et al., 2017 [24]	IL-8, IL-6, TNF-α	ELISA (Millipore)	IL-8 (pg/mL) 1614** IL-6 (pg/mL) 29.28** TNF-α (pg/mL) 41.12**	IL-8 (pg/mL) 485* IL-6 (pg/mL) 3.74* TNF-α (pg/mL) 11.12*	< 0.001*	↑ IL-8, IL-6, and TNF-α levels in group with DC compared to control group. A positive correlation was also observed between the three cytokines
Seyedmajidi et al., 2015 [25]	IL-8	ELISA (Reader)	IL-8 (pg/mL) 76.44	IL-8 (pg/mL) 86.4	> 0.05	↓ IL-8 levels in group with DC compared to control group
Gornowicz et al., 2012 [26]	IL-8, IL-6, TNF-α	ELISA (R&D Systems)	IL-8 (pg/mL) 1489.24(960.32)** IL-6 (pg/mL) 18.50(27.72)** TNF-α (pg/mL) 36.50(41.46)**	IL-8 (pg/mL) 619.19(311.79)* IL-6 (pg/mL) 2.68(5.51)* TNF-α (pg/mL) 7.32(6.98)*	< 0.008* < 0.005* < 0.002*	↑ IL-8, IL-6, and TNF-α levels in group with DC compared to control group.

Abbreviations: ELISA: Enzyme linked immunoabsorbent assay, IL-8: Interleukin-8, IL-6: Interleukin-6, TNF-α: Tumor necrosis factor- alpha, DC: Dental caries; NI: No information, NS: No significant

### Assessment of the quality of included studies and risk of bias

Regarding the cross-sectional studies and according to the established criteria, five (33%) [14, 18, 20, 21, 25] presented moderate bias, nine (60%) articles [12, 15–17, 19, 22–24, 26] presented moderate bias (Table 4). On the other hand, the only cohort study (7%) [13] had a low risk of bias (Table 5).

**Table 4:** JBI: risk of bias assessment of 14 included cross-sectional studies

Criteria →	1	2	3	4	5	6	7	8	Risk of bias score
Author’s/Year									
Nireeksha et al., 2024 [12]	X	X	X	X	X		X	X	**Low**
Leme et al., 2022 [14]	X	X	X	X			X	X	**Moderate**
Børsting et al., 2022 [15]	X	X	X	X	X		X	X	**Low**
Rinderknecht et al., 2022 [16]	X	X	X	X	X		X	X	**Low**
Prasanna et al., 2022 [17]	X	X	X	X	X		X	X	**Low**
AL-Dahhan et al., 2021 [18]	X	X	X	X			X	X	**Moderate**
Mohammed et al., 2021 [19]	X	X	X	X	X		X	X	**Low**
Ramírez-De los Santos et al., 2020 [20]	X	X	X	X			X	X	**Moderate**
Lo Giudice et al., 2019 [21]	X	X	X	X			X	X	**Moderate**
Nazemisalman et al., 2018 [22]	X	X	X	X	X		X	X	**Low**
Ribeiro et al., 2017 [23]	X	X	X	X	X		X	X	**Low**
Sharma et al., 2017 [24]	X	X	X	X	X		X	X	**Low**
Seyedmajidi et al., 2015 [25]	X	X	X	X			X	X	**Moderate**
Gornowicz et al., 2012 [26]	X	X	X	X	X		X	X	**Low**

X: Presence of criteria

(1)Were the criteria for inclusion in the sample clearly defined? (2)Were the study subjects and the setting described in detail? (3)Was the exposure measured in a valid and reliable way? (4)Were objective, standard criteria used for measurement of the condition? (5)Was confounding factors identified? (6)Were strategies to ideal with confounding factors stated? (7)Were the outcomes measured in a valid and reliable way? (8)Was appropriate statistical analysis used?

**Table 5:** JBI: risk of bias assessment of included cohort study

Questions →	1	2	3	4	5	6	7	8	9	10	11	Risk of bias score
Author’s/Year												
Biria et al., 2023 [13]	X	X	X	X		X	X	X	X	X	X	**Low**

X: Presence of criteria

Questions:

1 = Were the two groups similar and recruited from the same population?

2 = Were the exposures measured similarly to assign people to both exposed and unexposed groups?

3 = Was the exposure measured in a valid and reliable way?

4 = Were confounding factors identified?

5 = Were strategies to ideal with confounding factors stated?

6 = Were the groups/participants free of the outcome at the start of the study (or at the momento of exposure)?

7 = Were the outcomes measured in a valid and reliable way?

8 = Was the follow up time reported and sufficient to be long enough for outcomes to occur?

9 = Was follow up complete, and if not, were the reasons to loss to follow up described and explored?

10 = Were strategies to address incomplete follow up utilized?

11 = Was appropriate statistical analysis used?

### Clinical evidence comparing TNF-α, IL-6 and IL-8 levels in the exposure vs. control group: results of meta-analysis

**Fig. 2:**
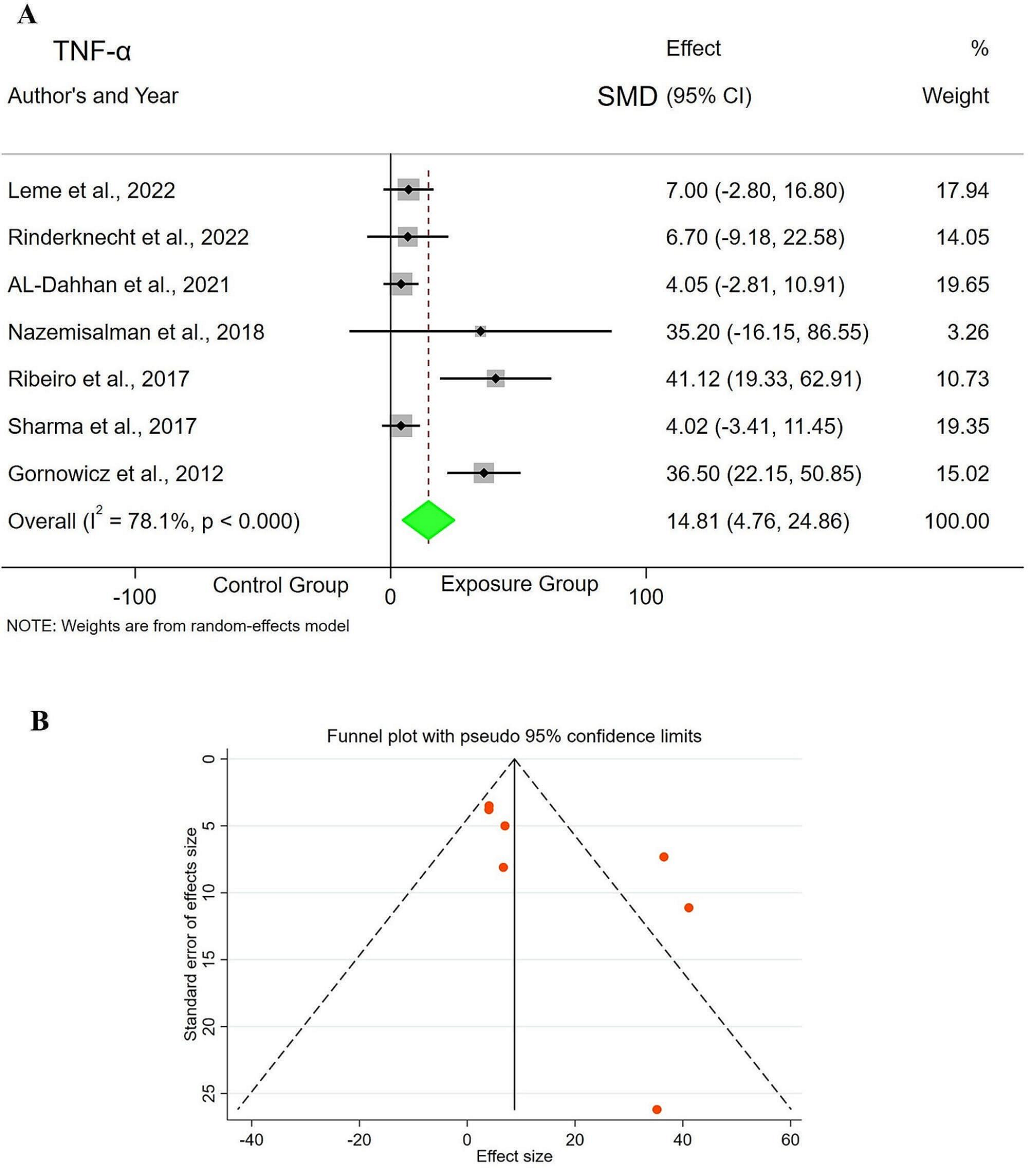
Forest plot comparing the TNF-α levels of **A**) Control Group vs. Exposure Group. **B**) Funnel plot to check the publication bias

Seven studies [14, 16, 18, 22–24, 26] compared saliva TNF-α levels between exposure group (*n* = 191) and control group (*n* = 157). The results of the meta-analysis indicated SMD = 14.81pg/ml (CI = 4.76–24.86, *p* = 0.004), demonstrating that TNF-α levels in saliva of children and young adults with moderate/severe active DC were significantly higher compared to the control group. Based on the chi-square test, there was evidence of heterogeneity among the studies (I2 = 78.1%, *p* = 0.000), noting that, the heterogeneity of the studies was relatively high, therefore a random effects model was used to pool the primary results. The funnel plot shows the asymmetry and possibility of publication bias. However, Egger’s test (t = 2.21, *p* = 0.079) showed no evidence of bias (Fig. 2).

**Fig. 3:**
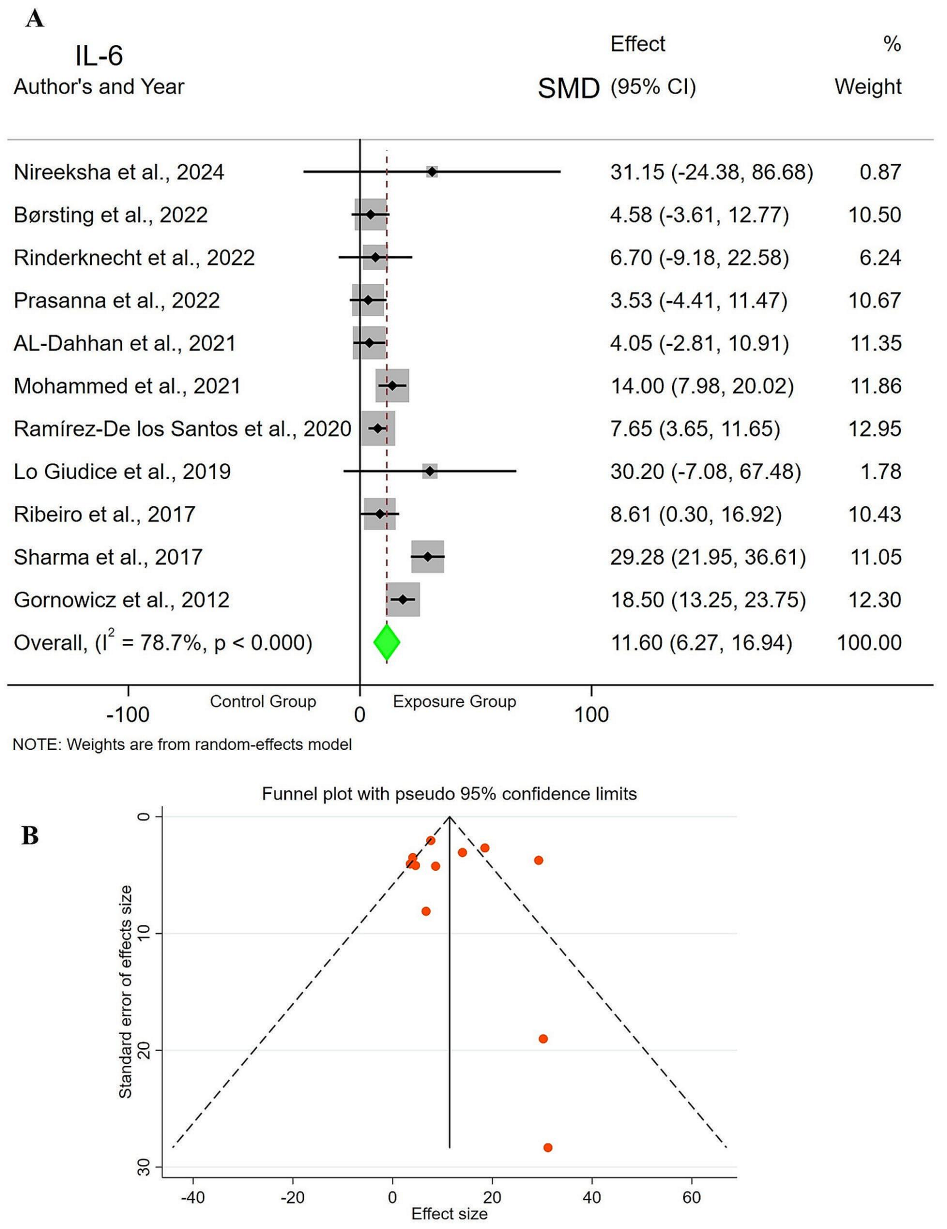
Forest plot comparing the IL-6 levels of **A**) Control Group vs. Exposure Group. **B**) Funnel plot to check the publication bias

Eleven studies [12, 14–20, 22, 23, 25] compared saliva IL-6 levels between the exposure group (*n* = 584) and control group (*n* = 295). The results of the meta-analysis indicated SMD = 11.60pg/ml (CI = 6.27–16.94, *p* = 0.000), demonstrating that the saliva IL-6 levels of children and young adults with active DC were significantly higher compared to the control group. Based on the chi-square test, there was evidence of heterogeneity among the studies (I2 = 78.7%, *p* = 0.000), noting that, the heterogeneity of the studies was relatively high, therefore a random effects model was used to pool the primary results. The funnel plot shows the asymmetry and possibility of publication bias. However, Egger’s test (t = 0.753, *p* = 0.753) showed no evidence of bias (Fig. 3).

**Fig. 4:**
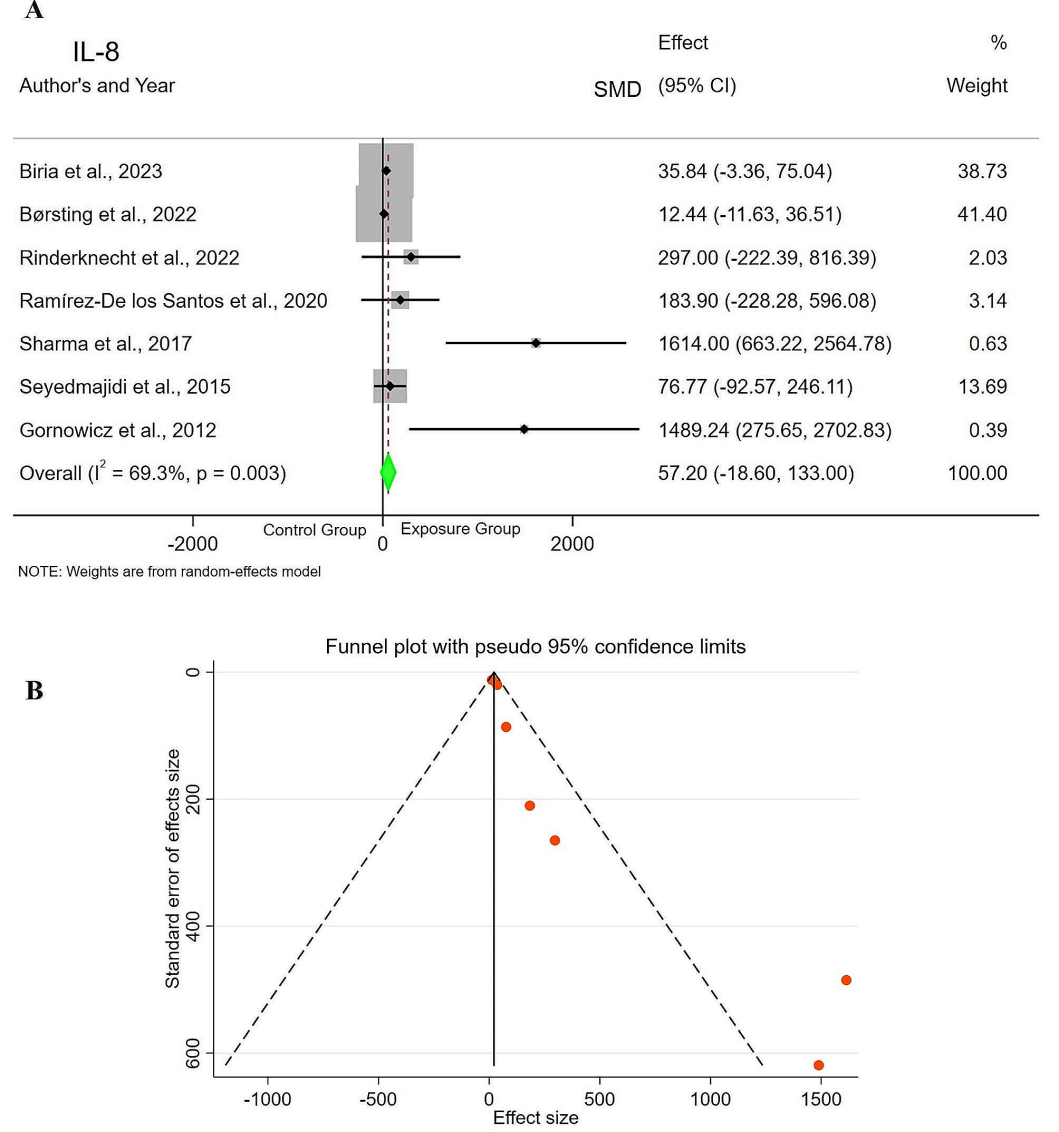
Forest plot comparing the IL-8 levels of **A**) Control Group vs. Exposure Group. **B**) Funnel plot to check the publication bias

Seven studies [13, 15, 16, 20, 24–26]compared saliva IL-8 levels between the exposure group (*n* = 349) and control group (*n* = 173). The results of the meta-analysis indicated SMD = 57.20pg/ml (CI=-18.60-133.00, *p* = 0.139), showing that the levels of IL-8 in saliva of children and young adults with active DC were higher compared to the control group, but without statistical significance. Based on the chi-square test, there was evidence of heterogeneity among the studies (I2 = 69.3%, *p* = 0.003), noting that, the heterogeneity of the studies was relatively high, therefore a random effects model was used to pool the primary results. The funnel plot shows the asymmetry and possibility of publication bias. In this case, Egger’s test (t = 4.23, *p* = 0.008) showed evidence of bias (Fig. 4).

## Discussion

**Fig. 5:**
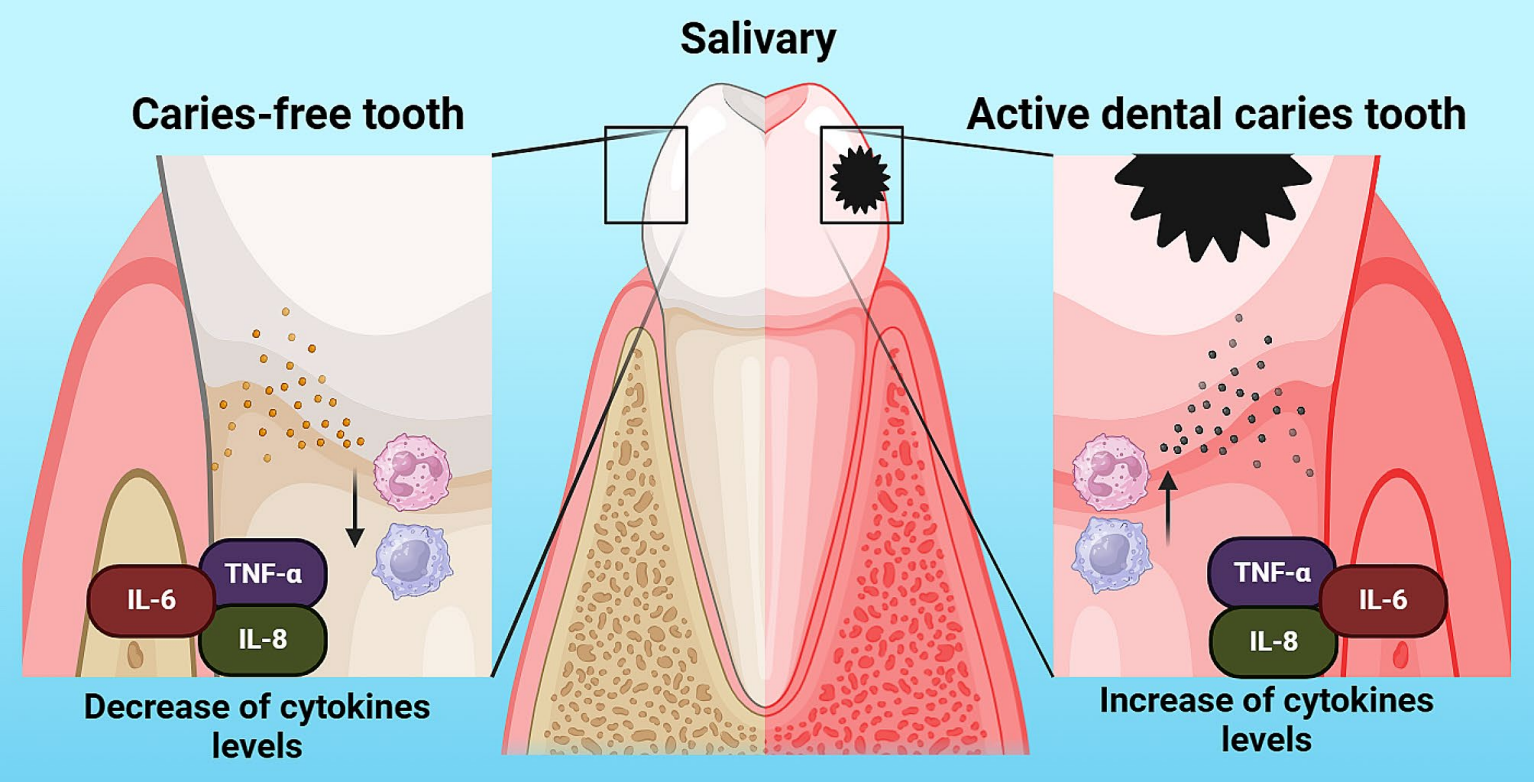
The levels of TNF-α, IL-6 and IL-8 increase in the saliva of teeth with active dental caries compared to teeth free of dental caries

A systematic review with a subsequent meta-analysis was carried out to evaluate and compare the levels of TNF-α, IL-6 and IL-8 in the saliva of children and young adults with active DC and control groups. A total of 14 cross-sectional studies and one cohort study were analyzed, which were conducted in nine different countries. Despite the high heterogeneity found, the main findings were the lack of articles that investigated other specific inflammatory and tissue destruction biomarkers in saliva; in fact, only two studies [15, 16] evaluated a broader panel of cytokines and chemokines, but a quantitative analysis of each of them could not be performed due to lack of information. Therefore, meta-analysis was only possible with these three inflammatory molecules, which are key in the pathogenesis of DC. Although some research [16–18, 20, 25] suggested a decrease in TNF-α, IL-6 and IL-8 levels, no statistically significant differences were found in those studies. However, quantitative analysis based on thirteen articles [12, 15, 16, 18–23, 25] suggested an increase in the levels of these cytokines in the exposure group compared to the control group. In the case of IL-8 no statistical significance was found. Cytokines play an important role in both innate and adaptive immune responses by modulating different mechanisms such as promotion, inhibition, recruitment, activation and cell differentiation [9, 10, 46, 47]. IL-6 is the most prevalent cytokine in saliva of children and young adults with DC, followed by TNF-α and IL-8, which was consistent with our results and with what has been published in the literature [12, 14–20, 22, 23, 25] (Fig. 5). For didactic purposes, the present discussion was divided into subtopics, which are presented below.

### An overview of DC and associated risk factors

DC corresponds to a chronic, complex and multifactorial infectious process that affects the tissues that constitute the crown and roots of teeth in both dentitions throughout their development [48]. It has a high prevalence worldwide (being more common in children and adolescents aged 5 to 17 years) [2, 3] and together with periodontitis, represents one of the main causes of dental abscess formation, pain and tooth loss [49]. Additionally, a greater association of DC has been reported in those individuals with metabolic diseases such as obesity [50], and diabetes [51], as well as in subjects with rheumatoid arthritis [52], and other systemic conditions. This is attributed to the fact that, involvement of the root canals and/or adjacent periodontium by the oral microbiota is likely to be one of the most direct pathways to the systemic circulation and thus may affect the target tissue [53]. Typically, like periodontitis, it is a disease mediated by the formation of polymicrobial dysbiotic biofilms, thus involving the presence of acidouric microorganisms that are in contact with the tooth surface, leading to a process of continuous demineralization [54]. In addition, other host factors include a deficiency in buffering capacity and salivary flow, as well as insufficient exposure to fluoride during the development of the dentition, poor oral hygiene conditions, poor maternal education together with inadequate forms of infant feeding, for example the use of a high sugar content in the diet, as well as nutritional alterations, such as vitamin D deficiencies [55, 56]. On the other hand, genetic factors that have been associated with this disease are the presence of SNPs in genes responsible for tooth formation (*AMELX* and *AQP5*), saliva formation and composition (*CA6*), those involved in carbohydrate metabolism (*TAS1R2* and *GLUT2*), and in immune responses (*IL-10*, *IL-1B* and *IL-6*) [57, 58]. Finally, in addition to a deprived lifestyle, low socioeconomic status increases the risk of DC in low- and middle-income countries. Therefore, the prognosis of the disease is closely related to oral health and the genetic and environmental risk factors that the patient may present. In this sense, it is essential to follow the following recommendations to reduce its prevalence in these settings. On the one hand, it is important to 1) limit highly cariogenic foods (sugars, snacks, juices), 2)improve education in the patient and their relatives about oral health, 3)incorporate national fluoride exposure programs to individuals at early ages, and 4)take into account sociodemographic limitations [59].

### Methods of sample collection and immunoassays

Clinical visual-tactile and visual-radiographic evaluation is essential for the diagnosis of DC [60]. However, with the current use of salivary biomarkers, the aim is to contribute to the prediction of the onset of the disease, as well as to monitor the progression of carious lesions [39–43].

Prior to immunoassay for the determination of inflammatory mediators, saliva samples can be collected in a stimulated manner by chewing a piece of gum or a piece of kerosene, or in an unstimulated manner by a passive drooling method or by asking the patient to spitting into a collection bottle, this over a time range of 5–10 min [61]. Normally, all saliva samples are collected between 9am and 11am in order to minimize the circadian rhythm in its composition. Once the sample is collected, it should be kept in cold net between 2 and 8 °C, and then transported to the laboratory as soon as possible for processing, or otherwise stored directly in a deep freezer at -80 °C [62]. After the centrifugation and supernatant recovery steps, the immunoassay technique that continues to be used most frequently today is the ELISA technique due to its high specificity and sensitivity [63].

### Salivary inflammatory biomarkers most frequently used in the diagnosis and/or prediction of DC

The inflammatory mediators that have been most frequently used to know and evaluate the dynamics of inflammation in DC are cytokines such as IFN-γ, IL-1β, IL-1α, TNF-α, IL-4, IL-5, IL-6, IL-7, IL-8, IL-10, IL-12B, IL-13, IL-15, IL-17, IL-18, OPG, LIFR, IL10RB, IL18R1, TNFRSF9, CSF1, TRAIL, TNFSF14, TRANCE, Fit3L LIF, TWEAK. And chemokines such as IL-8, MCP1, CXCL11, CXCL9, CXCL1, CCL4, SCF, CCL19, CXCL5, CCL23, CCL3, CXCL6, CXCL10, CCL28, MCP2, CX3CL1, CCL20, CD40. The most frequent were IL-6, TNF-α and IL-8 which again justifies the present meta-analysis [12–26].

### Effects of DC on the activity and levels of inflammatory cytokines

In this clinical scenario, saliva being in continuous and direct contact with the teeth represents one of the first lines of defense against cariogenic bacteria, because it presents several immunological mechanisms such as the production of antibodies (secretory IgA) [64], cytokines (IL-1β, TNF-α and IL-6) and chemokines (IL-8) [65] that are essential to control this disease [66]. Salivary IgA is considered a double-edged sword, since on the one hand, it neutralizes bacteria due to its dimeric arrangement contributing to their elimination, and on the other hand, it can also bind to salivary mucins, such as MUC1, favoring bacterial adhesion [67]. Regarding its role with respect to DC, a meta-analysis reported the presence of elevated levels of secretory IgA in saliva of subjects with active DC [68]. However, contradictorily, a more recent systematic review demonstrated that individuals free of carious lesions experienced an increased concentration of salivary IgA, suggesting that a reduction of this Ig increases the susceptibility to develop DC [40]. Therefore, this point is still unclear, there are still controversies regarding the concentrations/levels of secretory IgA in the saliva of subjects with DC, which is why, no definitive conclusions can be drawn that would allow labeling this protein as a potential salivary biomarker [41]. On the other hand, IL-1β is a proinflammatory, pleiotropic cytokine that acts by amplifying immune responses through the regulation of different mechanisms such as the promotion of myeloid cells, the differentiation of Th1, Th2 and Th17 cells, as well as the up-regulation of other cytokines/chemokines. It is a molecule that can produce vasodilation and chemotaxis of inflammatory cells, also induces collagen degradation by regulating the secretion of matrix metalloproteases (MMPs) and bone resorption by increasing osteoclastogenesis [69]. A positive association between the levels of these cytokines and DC has been demonstrated [70]. In fact, one study demonstrated an increase in IL-1β concentrations in saliva of 6- to 12-year-old children with moderate/severe carious lesions compared to their healthy controls [71]. Although on the other hand, Seyedmajidi et al., [25] found inverse levels of both IL-1β and IL-8, but without statistical significance. Therefore, the behavior of this cytokine is also unclear, and we encourage researchers to conduct future and follow-up studies to try to clarify these results.

In the case of TNF-α, Leme et al., 2022 [14], AL-Dahhan et al., 2021 [18], Nazemisalman et al., 2018 [22], Ribeiro et al., 2017 [23], Sharma et al., 2017 [24] and Gornowicz et al., 2012 [26] demonstrated that the levels of this cytokine in saliva of children and young adults with DC were increased and also associated with disease progression and severity. Only one study [16] found elevated levels of this cytokine in the control group compared to those with DC. Very similarly, Nireeksha et al., 2024 [12], Børsting et al., 2022 [15], Rinderknecht et al., 2022 [16], AL-Dahhan et al., 2021 [18], Mohammed et al., 2021 [19], Ramírez-De los Santos et al., 2020 [20], Lo Giudice et al., 2019 [21], Ribeiro et al., 2017 [23], Sharma et al., 2017 [24] and Gornowicz et al., 2012 [26] found higher IL-6 concentrations in children and young adults with active DC compared to their control groups. Only one study [17] found opposite levels. Likewise, regarding IL-8, Biria et al., 2023 [13], Børsting et al., 2022 [15], Rinderknecht et al., 2022 [16], Sharma et al., 2017 [24] and Gornowicz et al., 2012 [26] found increased levels of IL-8 in conditions of active DC compared to their control groups. In this case, only two studies [20, 25] demonstrated the opposite.

The exact role of these cytokines in DC has not been fully elucidated. However, this disease is closely related to the formation of dysbiotic biofilms, which are initially in contact with enamel and later, as they demineralize hydroxyapatite crystals come into contact with dentin and pulp tissue [72]. At these sites, bacteria use different mechanisms to evade the immune system causing inflammation and tissue damage [73]. But what happens at the level of dental tissues? On the one hand, odontoblasts, due to their arrangement at the periphery of the pulp chamber, constitute the first line of defense against bacterial challenge [74]. These cells, together with pulpal fibroblasts and adjacent immune cells such as dendritic cells, macrophages and salivary neutrophils express different pattern recognition receptors (PRRs) such as Toll-like receptors (TLR), mainly TLR1/2, TLR2/6, TLR3, TLR4, TLR5 and TLR9, as well as NOD-like receptors (NLR), such as NOD-2. Pathogen-associated molecular patterns (PAMPs) such as lipopolysaccharide (LPS), triacetylated lipopeptides, flagellin, lipoteichoic acid, viral dsRNA, and unmethylated DNA containing CpG motifs interact and bind with these receptors, leading to activation of the nuclear factor-κB (NF-κB) and mitogen-activated protein kinase (MAPK/p38) pathway [75]. In this case, NF-κB translocates from the cytoplasm to the nucleus and regulates the expression of proinflammatory genes, which are later translated into cytokines and chemokines (IL-1, IL-6 and IL-8) [76]. These cytokines participate in multiple processes such as chemotaxis, recruiting more phagocytes to the site of injury, also, they up-regulate the production of MMPs that contribute in the degradation of the extracellular matrix, induce osteoclast precursor cell differentiation, influence antibody production, and also induce the production of antimicrobial peptides (APs) such as beta-defensins (BD), which kill pathogens by altering their membrane integrity [77]. These APs such as BD1, BD2 and BD3 can also induce cytokine production in different cell types, creating a vicious positive feedback loop that accelerates disease development [78]. Therefore, the increased production of these inflammatory mediators would reflect one of the first host responses to polymicrobial challenge, as occurs in DC [79].

### Are TNF-α, IL-6 and IL-8 potential salivary biomarkers for assessing the severity of DC?

Although it is too early to define them punctually as inflammatory salivary biomarkers in the DC process, based on the available scientific evidence, it is presumed that, mainly IL-6 and TNF-α levels could be the potential to be considered as complementary tools to clinical and radiographic diagnosis to assess the severity of DC. However, further large-scale studies are still needed for the validation of these cytokines.

### Limitations

One of the main central strengths of the present work was the large sample size of 1,148 individuals. In addition, the use of the PECOS items ensured that only highly reliable research was selected and included, which ensured that the information analyzed was of high quality to increase the reliability of the review. Despite this, the present study also showed some limitations such as the inclusion of 14 cross-sectional studies and only one cohort study, which showed changes in the levels of these inflammatory cytokines after complete rehabilitation of carious lesions. The presence of high heterogeneity among study participants with respect to age and gender was another factor to be taken into account. Differences in the caries indices previously used, as well as the type of saliva and method of collection along with the immunoassay technique employed contributed to a wide variation in the results obtained that could extend to the inferences of the study.

## Conclusions

Based on the qualitative and quantitative analysis of the 15 studies included in the present review, the following can be concluded:

- IL-6 is the most prevalent cytokine in saliva of children and young adults with active DC, followed by TNF-α and IL-8.
- The most frequently used caries index was DMFT.
- The type of saliva collected most frequently was unstimulated saliva using the “Spitting” technique and for an average time of 8 min. In addition, in most cases, between 9am and 11am the collection process was carried out with an average of 3.89 mL of saliva.
- The ELISA technique was the immunoassay most used by the researchers, followed by the multiplex method.
- According to the results of the meta-analysis, a significant increase in the levels of IL-6 and TNF-α was found. These cytokines may have potential as complementary biomarkers to assess the severity of DC.

## Data Availability

The data supporting this study’s findings are available from the corresponding author upon reasonable request.

## Abbreviations

IFN-γ: Interferon gamma
IL-1β: Interleukin- 1 beta
IL-1α: Interleukin- 1 alpha
TNF-α: Tumor necrosis factor- alpha
IL-4: Interleukin- 4
IL-5: Interleukin- 5
IL-6: Interleukin- 6
IL-7: Interleukin- 7
IL-8: Interleukin- 8
IL-10: Interleukin- 10
IL-12B: Interleukin- 12B
IL-13: Interleukin- 13
IL-15: Interleukin- 15
IL-17: Interleukin- 17
IL-18: Interleukin- 18
OPG: Osteprotegerin
LIFR: Leukemia inhibitory factor receptor
IL10RB: Interleukin 10 receptor subunit beta
IL18R1: Interleukin 18 receptor 1
TNFRSF9: Receptor superfamily member 9
CSF1: Macrophage colony-stimulating factor
TRAIL: TNF-related apoptosis inducing ligand
TNFSF14: TNF superfamily member 14
TRANCE: TNF-related activation induced cytokine
FLT3LG: Fms-realted tyrosine kinase 3 ligand
LIF: Leukemia inhibitory factor
TWEAK: TNF ligand superfamily member 12
IL-8: Interleukin- 8
MCP1: Monocyte chemoattractant protein-1
CXCL11: C-X-C motif chemokine 11
CXCL9: C-X-C motif chemokine 9
CXCL1: C-X-C motif chemokine 1
CCL4: C-C motif ligands 4
SCF: Skp, Cullin, F-box containing complex
CCL19: C-C motif chemokine 19
CXCL5: C-X-C motif chemokine 5
CCL23: C-C motif ligands 23
CCL3: C-C motif ligands 3
CXCL6: C-X-C motif chemokine 6
CXCL10: C-X-C motif chemokine 10
CCL28: C-C motif ligands 28
MCP2: Monocyte chemoattractant protein-2
CX3CL1: C-X3-C motif ligand 1
CCL20: C-C motif ligands 20
CD40: Cluster of differentiation 40
